# Supplementary material for: Investigating the effect of climate and air pollution on prescription uptake in the England
Source: BMC Public Health. 2026 Jun 11;26:2770. doi: 10.1186/s12889-026-28114-5 (PMC13637115; doi:10.1186/s12889-026-28114-5)
Supplement: Supplementary file 1 [file 12889_2026_28114_MOESM1_ESM.pdf]

Supplementary Information for *Investigating the Effect of Climate and Air Pollution on Prescription Uptake in England*

John Tolladay[1] and Christopher Yau*[1,2]

[1]Nuffield Department of Women's and Reproductive Health, University of Oxford, Oxford, United Kingdom
[2]Health Data Research UK, London, UK

Contents

S1 Distributional diagnostics for preprocessing **1**
S1.1 Practices and observations affected 1
S1.2 Distributional summary 1
S1.3 Examples of practice-level cleaning 3

S2 Sensitivity to the COVID-19 period **3**

S3 Percentage change in prescription volumes with up to two months lag **4**

S1 Distributional diagnostics for preprocessing

The main text describes three preprocessing rules applied to the practice-month prescribing dataset: (i) exclusion of practices with mean monthly items ≤ 10; (ii) within each remaining practice, removal of months with item counts below 10% of the practice-specific mean; and (iii) retention only of practices with at least 20 monthly observations after rules (i) and (ii). This section documents the distributional impact of these rules.

S1.1 Practices and observations affected

Table S1 reports the proportion of practices and month-practice observations affected by each rule individually and in combination. Although the rules together drop 54.6% of practices, these are predominantly very low-volume practices and the affected observations represent only 8.3% of all month-practice datapoints. The cleaned dataset retains 10,155 practices contributing 1,396,426 monthly observations.

S1.2 Distributional summary

Table S2 compares the raw and cleaned datasets across summary statistics and percentiles. Cleaning shifts the mean and lower percentiles upward (because very low-volume practices

*Corresponding author: christopher.yau@wrh.ox.ac.uk

Rule	Practices affected n	%	Month-practice datapoints n	%
Practice mean ≤ 10	4,330	28.55	96,607	6.34
Month $\leq \max($practice mean$/10, 1)$	3,776	24.90	23,475	1.54
$<$ 20 months after first 2 rules	681	4.49	6,675	0.44
Any rule (combined)	8,282	54.61	126,757	8.32
Raw dataset	15,166	100.00	1,523,183	100.00
Clean dataset	10,155	66.96	1,396,426	91.68

Table S1: Practices and month-practice observations removed by each preprocessing rule. Rules are applied sequentially in the order shown. The combined "Any rule" line is not the sum of the individual lines because some practices are caught by more than one rule.

are removed) but leaves the upper percentiles and overall variability essentially unchanged, indicating that cleaning removes a low-count tail without altering the bulk of the distribution. Figure S1 shows this graphically: the cleaned distribution is a near-perfect overlay of the raw distribution above ~100 monthly items, with a low-count tail removed below this threshold.

Group	Coverage practices	datapoints	Summary mean	std	Percentiles 25	50	75	95	100
Raw dataset	15,166	1,523,183	4,079	3,768	1,439	3,323	5,828	10,791	66,927
Clean dataset	10,155	1,396,426	4,446	3,723	1,920	3,676	6,103	11,073	66,927

Table S2: Distributional summary of monthly prescription items, raw vs. cleaned datasets.

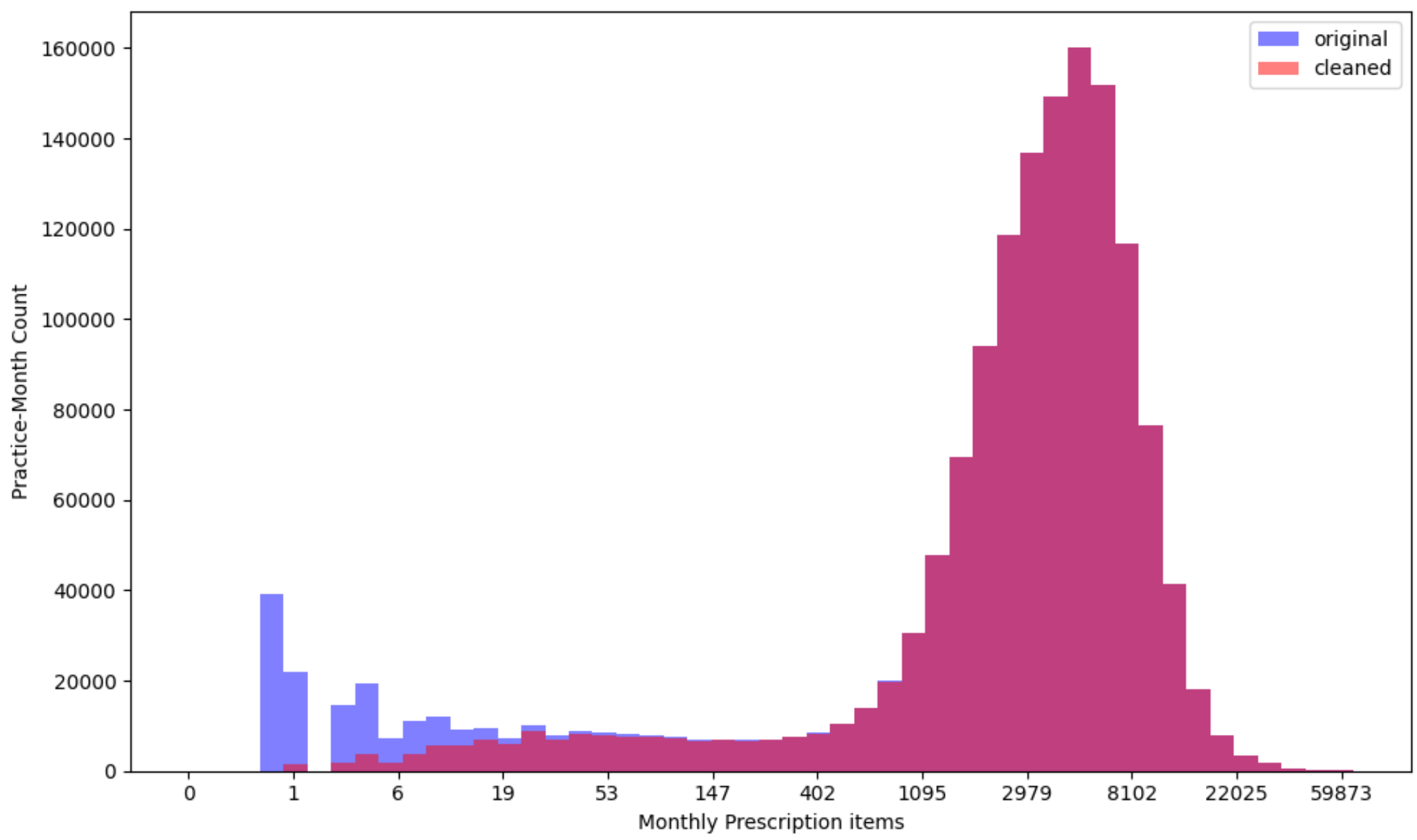

Figure S1: Distributions of monthly prescription counts on a log scale for the raw (blue) and cleaned (red) datasets. Cleaning removes a low-count tail without altering the bulk of the distribution.

S1.3 Examples of practice-level cleaning

Figure S2 shows monthly prescription time-series for twenty randomly selected practices, with retained datapoints shown as red crosses overlaid on the original blue circles. Panels marked "Dropped" correspond to practices excluded by either the ≤ 10 mean-volume rule or the < 20-month coverage rule. The dropped panels are characterised by long stretches of zero or near-zero counts inconsistent with active prescribing (e.g. Y04680, Y07757, Y00749, Y05821, Y06436, Y05085, Y00706, Y00323), while in retained practices the cleaning rules typically remove only isolated artefactual zero-months at the edges of the practice's coverage period without affecting the temporal structure of the series.

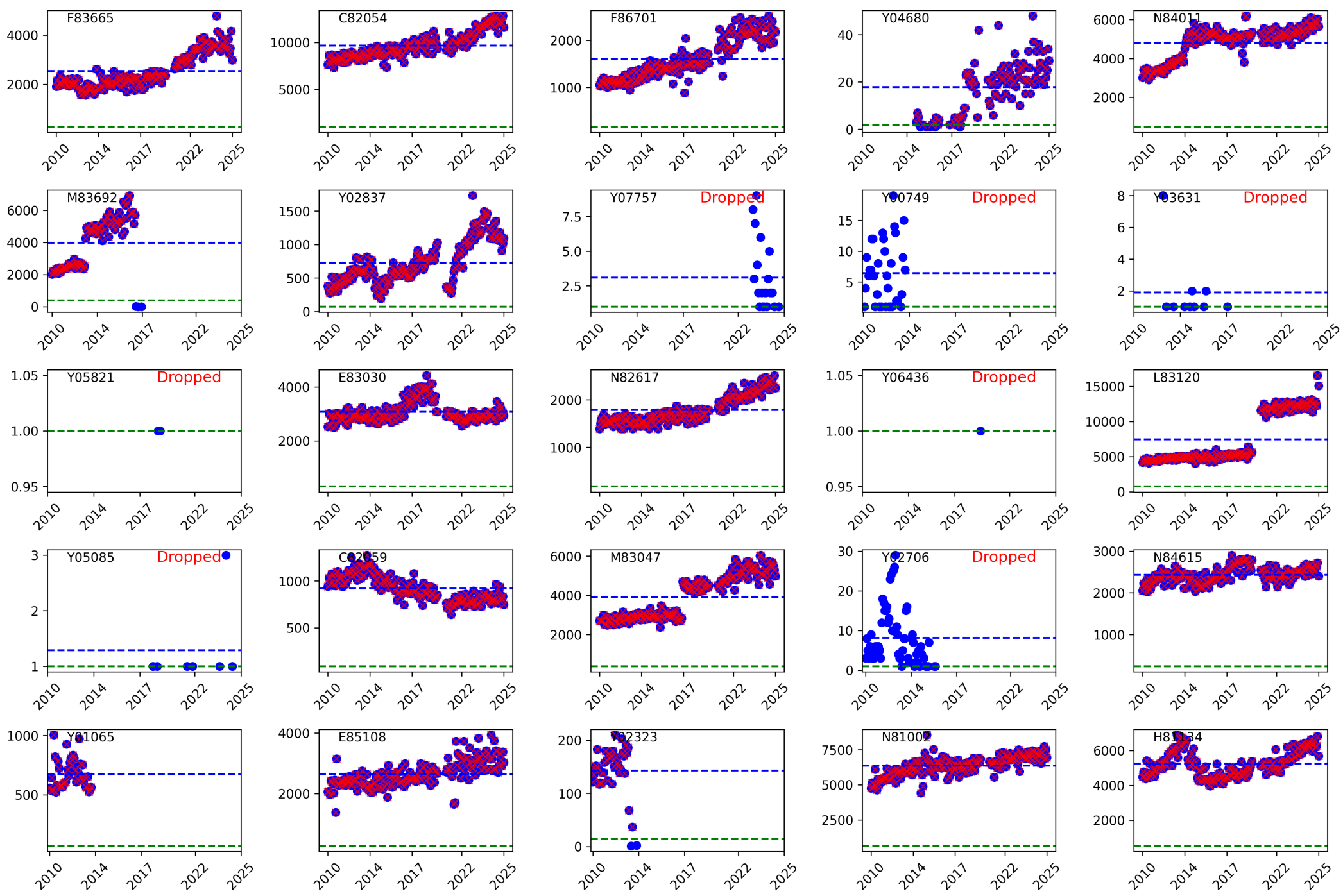

Figure S2: Monthly prescription volumes for twenty randomly selected practices. Blue circles are original datapoints; red crosses are retained after cleaning. Blue dashed lines indicate the practice mean and green dashed lines the 10% practice-specific cut-off. Panels labelled "Dropped" identify practices excluded by the ≤ 10 mean-volume or < 20-month coverage rules.

S2 Sensitivity to the COVID-19 period

To assess whether the COVID-19 period materially influenced our findings, we re-ran both the mixed-effects and Bayesian multivariate analyses using only data up to February 2020 ("pre-COVID model"), and compared the resulting estimates with those of the full model.

Table S3 compares the distribution of monthly prescription items across the pre-COVID period (data up to February 2020), the COVID period (March 2020 - March 2021 inclusive), and the post-COVID period (from April 2021 onwards). Mean and median item counts increased modestly during and after the pandemic but the overall shape of the distribution is preserved.

Group	Summary n	mean	std	Percentiles 25	50	75	95	100
Raw pre-COVID	1,032,951	3,788	3,317	1,436	3,107	5,450	9,843	58,348
Raw during COVID	57,012	4,381	4,070	1,579	3,646	6,187	11,522	56,122
Raw post-COVID	433,220	4,732	4,568	1,431	3,935	6,735	12,740	66,927
Clean pre-COVID	952,931	4,102	3,263	1,818	3,401	5,686	10,065	58,348
Clean during COVID	52,481	4,758	4,025	2,121	3,992	6,452	11,775	56,122
Clean post-COVID	391,014	5,241	4,522	2,276	4,408	7,111	13,125	66,927
Raw all months	1,523,183	4,079	3,768	1,439	3,323	5,828	10,791	66,927
Clean all months	1,396,426	4,446	3,723	1,920	3,676	6,103	11,073	66,927

Table S3: Distributional summary of monthly prescription items before, during (March 2020 - March 2021, inclusive), and after the COVID-19 period.

S3 Percentage change in prescription volumes with up to two months lag

We computed posterior estimates from the Bayesian multivariate model for percentage change in prescription volumes relative to standard deviation increase in predictors with up to two months lag. Figure S3 shows shading around each line giving the 95% equal-tailed credible intervals for nitrous oxides (NOx) and particulate matter (PM2.5 and PM10) $< 2.5\mu$m and $< 10\mu$m in diameter, respectively.

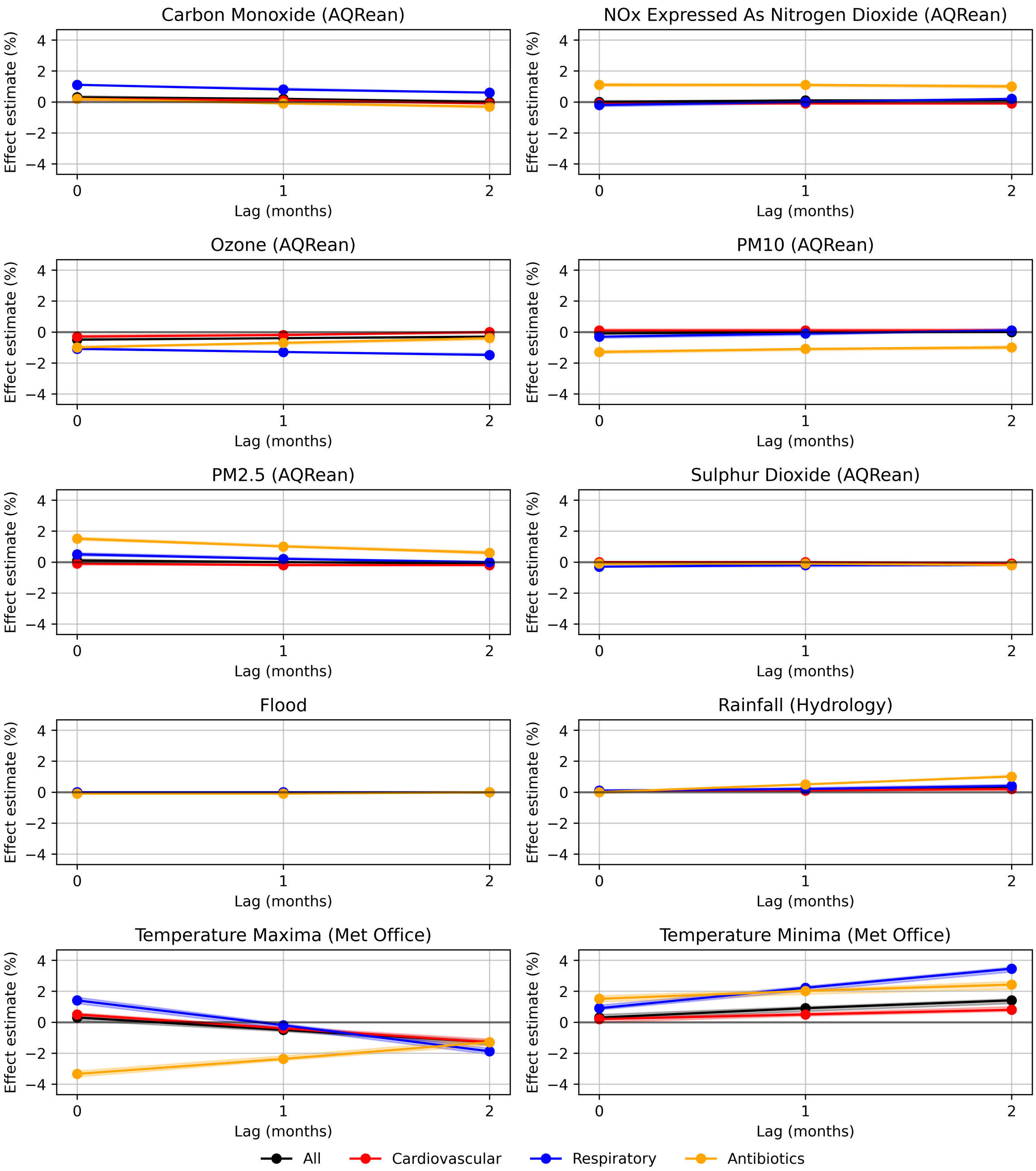

Figure S3: Posterior estimates from Bayesian multivariate model for percentage change in prescription volumes relative to standard deviation increase in predictors with up to two months lag. Shading around each line shows 95% equal-tailed credible intervals. NOx = nitrous oxides. PM2.5 and PM10 refer to particulate matter $< 2.5\mu$m and $< 10\mu$m in diameter, respectively.
